# Just watching the game ain't enough: striatal fMRI reward responses to successes and failures in a video game during active and vicarious playing

**DOI:** 10.3389/fnhum.2013.00278

**Published:** 2013-06-13

**Authors:** Jari Kätsyri, Riitta Hari, Niklas Ravaja, Lauri Nummenmaa

**Affiliations:** ^1^Department of Media Technology, Aalto University School of ScienceEspoo, Finland; ^2^School of Business, Aalto UniversityHelsinki, Finland; ^3^Brain Research Unit, O.V. Lounasmaa Laboratory, Aalto University School of ScienceEspoo, Finland; ^4^Advanced Magnetic Imaging Centre, Aalto NeuroImaging, Aalto UniversityEspoo, Finland; ^5^Helsinki Institute for Information Technology, Aalto UniversityEspoo, Finland; ^6^Department of Social Research, University of HelsinkiHelsinki, Finland; ^7^Department of Biomedical Engineering and Computational Science, Aalto University School of ScienceEspoo, Finland; ^8^Turku PET Centre, University of TurkuTurku, Finland

**Keywords:** emotion, motivation, natural stimulation, reward system, striatum, video-game playing

## Abstract

Although the multimodal stimulation provided by modern audiovisual video games is pleasing by itself, the rewarding nature of video game playing depends critically also on the players' active engagement in the gameplay. The extent to which active engagement influences dopaminergic brain reward circuit responses remains unsettled. Here we show that striatal reward circuit responses elicited by successes (wins) and failures (losses) in a video game are stronger during active than vicarious gameplay. Eleven healthy males both played a competitive first-person tank shooter game (active playing) and watched a pre-recorded gameplay video (vicarious playing) while their hemodynamic brain activation was measured with 3-tesla functional magnetic resonance imaging (fMRI). Wins and losses were paired with symmetrical monetary rewards and punishments during active and vicarious playing so that the external reward context remained identical during both conditions. Brain activation was stronger in the orbitomedial prefrontal cortex (omPFC) during winning than losing, both during active and vicarious playing. In contrast, both wins and losses suppressed activations in the midbrain and striatum during active playing; however, the striatal suppression, particularly in the anterior putamen, was more pronounced during loss than win events. Sensorimotor confounds related to joystick movements did not account for the results. Self-ratings indicated losing to be more unpleasant during active than vicarious playing. Our findings demonstrate striatum to be selectively sensitive to self-acquired rewards, in contrast to frontal components of the reward circuit that process both self-acquired and passively received rewards. We propose that the striatal responses to repeated acquisition of rewards that are contingent on game related successes contribute to the motivational pull of video-game playing.

## Introduction

Video game playing is intrinsically motivating (cf. Ryan and Deci, 2000): most people play video games because they are inherently interesting and enjoyable rather than because they provide financial rewards or other external outcomes (Ryan et al., 2006; Przybylski et al., 2009, 2010). Accordingly, brain imaging studies have demonstrated that video game playing engages key motivational systems of the brain, as evidenced by increases in dopamine release (Koepp et al., 1998) and hemodynamic activations (Hoeft et al., 2008) in the striatum (see also Kätsyri et al., 2012). Major motivational events during the gameplay consist of successes and failures to achieve specific game goals, such as managing to eliminate one's opponents or avoiding getting eliminated oneself. Successes and failures are among the most potent triggers for pleasant and unpleasant emotions (Nummenmaa and Niemi, 2004), and their affective salience is amplified when they can be attributed to internal (as during active gameplay) rather than external causes (Weiner, 1985). In line with this, brain imaging studies have shown that self-acquired rewards—such as those contingent on correct motor responses—rather than those delivered at random evoke stronger neural responses in the striatum (e.g., Zink et al., 2004). Consequently, it is possible that the motivational pull of video games could be explained by the amplified reward responses triggered by actively obtaining rewards during gameplay. Here we tested this hypothesis by contrasting reward circuit responses to success- and failure-related gameplay events during *active* and *vicarious* video-game playing—that is, situations in which players have complete versus no control over their game character.

Success- and failure-related gameplay events fulfil the three characteristics of rewards and punishments considered in animal learning (Schultz, 2004, 2006; Berridge and Kringelbach, 2008). First, they contribute to learning by providing direct feedback on the players' performance. Second, they are associated with approach and withdrawal behaviors, given that players strive to succeed and to avoid failing in the game (see Clarke and Duimering, 2006). Third, successes are generally associated with pleasant and failures with unpleasant emotional responses—even though in some games this mapping may be more complex (Ravaja et al., 2006, 2008). Dopaminergic pathways extending from the midbrain (ventral tegmental area and substantia nigra, VTA/SN) to the ventral and dorsal striatum (nucleus accumbens, caudate nucleus, and putamen) and frontal cortex (orbitomedial and medial prefrontal cortex; omPFC and vmPFC) are involved in processing rewards and punishments (Kelley, 2004; O'Doherty, 2004; Bressan and Crippa, 2005; Knutson and Cooper, 2005; Schultz, 2006; Berridge and Kringelbach, 2008; Hikosaka et al., 2008; Haber and Knutson, 2010; Koob and Volkow, 2010). This dopaminergic circuitry also likely contributes to encoding successes and failures during video-game playing. For example, neurons in monkey lateral PFC are differentially activated by successes and failures in a competitive shooting game (Hosokawa and Watanabe, 2012). Moreover, functional magnetic resonance imaging (fMRI) studies in humans have shown that successes in a video game evoke stronger activations than do failures in nucleus accumbens, caudate, and anterior putamen, as well as mPFC (Mathiak et al., 2011; Kätsyri et al., 2012; Klasen et al., 2012), and that the most anteroventral striatal activations correlate with the players' self-rated hedonic experiences during these events (Kätsyri et al., 2012).

The striatum is extensively connected to associative, motor, and limbic circuits, thereby being in an ideal anatomical position to combine both motor and affective information (Haber and Knutson, 2010). Both animal and human studies have consistently indicated that striatal reward responses are contingent on the rewards themselves as well as the actions performed to acquire them (cf. Delgado, 2007). Monkey caudate neurons fire more frequently during motor actions leading to expected rewards than during non-rewarded actions (Kawagoe et al., 1998; Schultz et al., 2000). Human fMRI studies have similarly demonstrated contingency between action and reward in the striatum. For example, dorsal caudate responds differentially to rewards and punishments only when they are perceived to be contingent on the participants' button presses (Tricomi et al., 2004). Similarly, reward activations in putamen are elevated only when the rewards are contingent on button presses (Elliott et al., 2004). Furthermore, activations in the whole striatum have been found for button presses that were executed to obtain rewards or to avoid punishments (Guitart-Masip et al., 2011). Particularly the ventral striatum shows increased activation after verbal feedback following successful motor performance, both in the absence and presence of monetary rewards (Lutz et al., 2012). Unlike the striatum, the omPFC processes reward independently of motor actions both in monkeys (Schultz et al., 2000) and humans (Elliott et al., 2004). Following these findings, successes should evoke stronger activations in the striatum than failures only during active video game playing, whereas the omPFC should show stronger activations to successes both during active and vicarious playing.

Up to date, few brain imaging studies have compared neural activations during active and vicarious video game playing. One study using electroencephalography demonstrated that active versus vicarious playing evokes increased fronto-parietal cortical activations, along with higher self-reported spatial presence in the game (Havranek et al., 2012). Haemodynamic responses to active and vicarious playing have been explicitly compared in only one study (Cole et al., 2012): the onset of video game activated the striatum (nucleus accumbens, caudate, and putamen) and frontal nodes adjacent to mPFC (i.e., anterior cingulate cortex), with stronger activation during active than vicarious gameplay. The striatal activations decreased following the offset of playing. However, the fMRI responses to success events in the game did not differ between active and vicarious playing; furthermore, failure events were not included in the game. It is possible that the applied between-subjects design (i.e., comparison between participant groups playing and watching a video game) was not powerful enough to reveal success-related differences between active and vicarious playing. Furthermore, the study naturally begs the question of whether failure events would evoke differential activations during active and vicarious gameplay.

Here we investigated whether the reward-system activations elicited by successes and failures in a competitive video game would differ between active and vicarious video-game playing in a fully within-subjects design. We used a simplified tank shooter game that was customized for the fMRI setting (cf. Kätsyri et al., 2012). The major success and failure events in the game consisted of wins (eliminating the opponent) and losses (getting eliminated oneself) against one's opponent. We reanalyzed parts of our previously published data on active gameplay (Kätsyri et al., 2012), and compared them with novel data from watching the same game. Unlike in our previous analysis of active gameplay data, we now contrasted win and loss events separately, given that recent evidence suggests that striatal activations decrease both during wins and losses during active gameplay (Mathiak et al., 2011). We paired win and loss events with symmetric monetary rewards and punishments during both active and vicarious playing, so that the external reward for wins and losses remained identical in both conditions. Based on the previous literature, we predicted that the striatum (particularly, nucleus accumbens, ventral caudate, and anterior putamen) would show a stronger difference between wins and losses during active than vicarious gameplay, and that these effects would be associated with corresponding amplified experiences of pleasant and unpleasant emotions. We also predicted that wins would evoke greater responses in the mPFC (in particular, omPFC) than losses both during active and vicarious playing, and that these differential activations would correlate with self-rated pleasantness and unpleasantness evaluations.

## Materials and methods

### Participants

The participants were eleven right-handed male volunteers with a mean age of 25.6 years (range 22–33 years) and with abundant experience in gaming (mean 7.8 h/week, range 1–20 h/week). Additional six participants were scanned but excluded from the analysis due to technical problems (one participant), deviant playing strategies (extensive button pressing; one participant), or excessive head movements (four participants). The total playing time reported by all participants was below 30 h/week, which is an often-used criterion for addictive video game playing (Ko et al., 2009; Han et al., 2010). None of the participants had prior experience of the game played in the present study. All but one participant reported playing first-person shooter games on a regular basis with modest weekly play times (mean 3.2 h/week, range 0.5–10 h/week). All participants were Finnish under- or post-graduate university students. Only male participants were recruited because men typically have more experience of video games, are generally more motivated by such games, and show higher preference than women for competitive video games (Lucas, 2004). Participants with self-reported history of neurological or psychiatric disorders were excluded. All participants provided written informed consent as part of a protocol approved by the Ethics Committee of the Helsinki and Uusimaa University District and received monetary compensation for their lost working hours.

### Statistical power

For statistical power calculations, we used previous active gameplay data (*N* = 43 participants) from Cole et al. (2012). Given that their experiment did not include explicit comparison between wins and losses during active versus vicarious gameplay, we instead adopted their reported statistics on NAcc responses to active gameplay onsets (*M* = 0.234 and *SD* = 0.2015). Next, using G^*^Power software (Faul et al., 2007), we estimated the *a priori* statistical power of the present experiment to detect similar effect sizes (γ = 0.234/0.2015 = 1.16). The estimated power was 93%, which was considered satisfactory for the present purposes.

### Experimental procedure

Our experimental setting has been described in detail previously (Kätsyri et al., 2012). Briefly, during scanning the participants played two sessions of a first-person tank-shooter game “BZFlag” (an in-house modified version of 2.0.14; http://bzflag.org) against alleged human and computer opponents, respectively, and watched one pre-recorded gameplay video. Sessions lasted 10 min each and were presented in a counterbalanced order. However, to avoid possible reward-response biases due to competing against another human (cf. Kätsyri et al., 2012), we here analyzed only the computer-opponent session. One participant whose video-watching data were missing was replaced with a new participant; otherwise, the computer-opponent data were identical to our previous data (Kätsyri et al., 2012).

**Figure 1:**
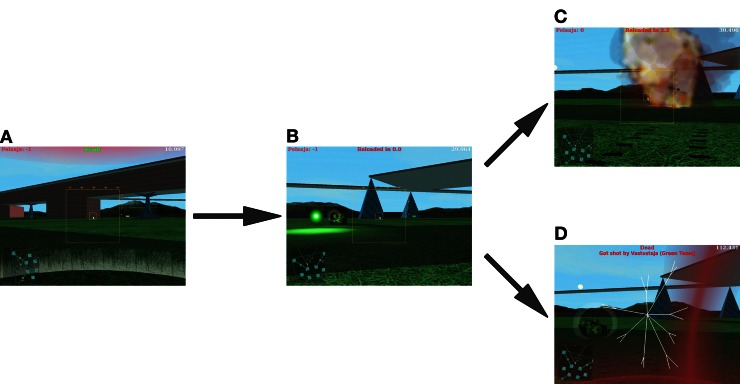
**A sample sequence of gameplay events: player spawns in a random location on the battlefield and starts searching for the opponent (A), the player and opponent engage each other (B), until the player either manages to eliminate the opponent (C) or gets eliminated himself (D)**.

Effects of active versus vicarious gameplay on win- and loss-related activations were evaluated in a 2 (win versus lose) ×2 (play vs. watch) within-subjects design. During active playing, the participant's task was to seek and destroy the opponent's tank from the battlefield without getting destroyed himself (Figure 1). The corresponding win and loss events, as well as joystick movements, were time-referenced to fMRI scans and logged automatically for statistical analyses. During vicarious gameplay, the participant's task was to follow a gameplay video recorded with the video capture software FRAPS (http://www.fraps.com) from one player, who did not take part in the actual study. Frequencies of wins and losses in the gameplay video were similar to those in the gameplay sessions (cf. Table 1). The final video had similar resolution (video: 1024 × 768 pixels sampled at 30 fps, audio sampled at 48 kHz) and visual quality (15 Mbit/s after video compression with XVID codec; http://www.xvid.com) as the actual video game. The gameplay video was presented using Presentation software (http://www.neurobs.com).

**Table 1:** **Mean ± SEM behavioral and self-report measures from active (playing) and vicarious (watching) video-game playing sessions**.

**Behavioral measure**	**Playing**	**Watching**	***P***
Game score	3.1 ± 1.5	3.0^a^	0.964
Wins	18.6 ± 1.2	20.0^a^	0.309
Losses	15.6 ± 1.2	17.0^a^	0.247
Game experience			
Challenge	3.0 ± 0.2	2.2 ± 0.3	0.057^†^
Competence	3.0 ± 0.3	2.6 ± 0.3	0.237
Flow	3.2 ± 0.3	2.3 ± 0.3	0.007^**^
Negative affect	1.4 ± 0.2	2.4 ± 0.3	0.014^*^
Positive affect	3.3 ± 0.3	2.6 ± 0.3	0.074^†^
Immersion	2.9 ± 0.2	2.2 ± 0.2	0.022^*^
Tension	1.9 ± 0.3	2.1 ± 0.3	0.469
Spatial presence	3.1 ± 0.2	2.4 ± 0.3	0.008^**^
Social presence			
Empathy	1.8 ± 0.2	1.7 ± 0.2	0.725
Involvement	3.8 ± 0.2	3.4 ± 0.3	0.139
Negative feelings	2.4 ± 0.3	2.2 ± 0.2	0.403
Pleasantness			
Win events	7.5 ± 0.2	7.0 ± 0.4	0.238
Loss events	2.8 ± 0.5	3.4 ± 0.3	0.034^*^

The self-rating measures were scored on a scale ranging from 1 to 5 except for pleasantness ratings, which ranged from 1 (most unpleasant) to 9 (most pleasant). Game scores refer to numbers of wins minus numbers of losses. Significance values are from Wilcoxon matched-pairs tests.

a Identical for all participants.

† P < 0.10.

* P < 0.05.

** P < 0.01.

To control for the external reward context for active and vicarious playing conditions, we introduced symmetric monetary rewards and punishments to wins and losses, respectively. Participants were told that in addition to a fixed compensation (20 Euros), they would gain money (+0.33 Euros) when winning and lose money (−0.33 Euros) when losing during the gameplay or when watching the player winning or losing on the video. In reality, all participants received an equal monetary compensation (30 Euros), which exceeded the sum any of them would otherwise have gained.

### Self-reports

Before the experiment, participants filled a 20-item self-evaluation questionnaire related to their dispositional behavioral inhibition and activation system (BIS/BAS) sensitivities (Carver and White, 1994). The BIS and BAS regulate aversive and appetitive motivation, modulating behavioral and affective responses towards punishments and rewards, respectively (Carver and White, 1994). The BIS scale is comprised of seven items (e.g., “I feel pretty worried or upset when I think or know somebody is angry at me”). The BAS scale is comprised of three subscales: drive (4 items; e.g., “I go out of my way to get things I want”), reward responsiveness (5 items; e.g., “When I get something I want, I feel excited and energized”), and fun seeking (4 items; e.g., “I crave excitement and new sensations”). Each of the items was rated on a 4-point scale, ranging from 1 (very false with me) to 4 (very true for me). The psychometric properties of the instrument have been shown to be acceptable (Carver and White, 1994).

To assess participants' subjective experiences during active and vicarious playing, we asked them to complete a series of self-reports after both gameplay sessions. The order of questions was randomized, and the responses were given by moving the joystick. We used the Game Experience Questionnaire (Ijsselsteijn et al., 2008) to quantify the following facets of gaming experience: challenge, competence, flow, positive affect, negative affect, immersion, and tension (two items per scale). Spatial presence—the experience of being physically present in the game environment (Lombard and Ditton, 1997)—was measured with the Spatial Presence scale of the ITC Sense of Presence Inventory (Lessiter et al., 2001). The Spatial Presence scale is comprised of 19 items (e.g., “I had a sense of being in the game scenes”). To measure the participants' experience of taking part in a social interaction with their opponent, we used the Social Presence in Gaming Questionnaire (de Kort et al., 2007), which consists of 17 items related to empathy (e.g., “I empathized with the other”), involvement with the other player's actions (e.g., “My actions depended on the other's actions”), and negative feelings towards him (e.g., “I felt revengeful”). Two additional questions were used for evaluating overall pleasantness of all win and all loss events during a session, on a scale ranging from 1 (extremely unpleasant) to 5 (neither pleasant nor unpleasant) to 9 (extremely pleasant).

### Joystick regressors

Horizontal and vertical joystick coordinates were digitized at 200 Hz and collapsed into Euclidean distances from the joystick's central position. Resulting position and velocity (i.e., the first derivative of position) tracks were low-pass filtered at 5 Hz using a first-order smoothing filter (Savitzky and Golay, 1964). Mean joystick position and velocity values were extracted separately for each fMRI scan of each participant. Finally, to remove any overlap between these time courses, the joystick velocity time course was orthogonalized with respect to the joystick position track. Consequently, the joystick position regressor measured the overall tank movement, whereas the joystick velocity regressor measured how much the player exerted control over the tank's movement during each fMRI scan. Similar regressors were extracted for the watching condition from the game logs of the player whose gameplay session was shown on the video. These variables were subsequently used as nuisance covariates in the fMRI data analysis.

### Acquisition and analysis of fMRI data

#### Data acquisition and preprocessing

Functional and anatomical volumes were collected with a General Electric Signa 3.0 T MRI scanner at the Advanced Magnetic Imaging Centre of Aalto University. Whole-brain functional images were acquired using weighted gradient-echo planar imaging, sensitive to BOLD signal contrast (35 oblique slices without gaps, slice thickness = 4 mm, TR = 2070 ms, TE = 32 ms, FOV = 220 mm, flip angle = 75°, interleaved slice acquisition, 293 volumes per session with a resolution of 3.4 × 3.4 mm^2^). The first three volumes were discarded to allow for equilibration effects. T1-weighted structural images were acquired at a resolution of 1 × 1 × 1 mm^3^ using a sequence with ASSET calibration.

Preprocessing and analysis of fMRI data were performed using SPM8 software package (Wellcome Department of Imaging Neuroscience, London) in Matlab (version 7.11). The EPI images were sinc interpolated in time to correct for slice timing differences and realigned to the first scan by rigid-body transformations to correct for head movements. ArtRepair toolbox (version 4; http://spnl.stanford.edu/tools/ArtRepair; Mazaika et al., 2009) was used to correct for movement artifacts. Realigned functional volumes were first motion-adjusted and outlier volumes (head position change exceeding 0.5 mm or global mean BOLD signal change exceeding 1.3%) were then replaced by linear interpolation between the closest non-outlier volumes. Four participants with more than 10% outlier volumes were removed from further analysis. On average, 2.5% of volumes during the video game playing session and 1.5% of volumes during the video game watching were classified as outliers—the number of outliers was not significantly different between these conditions (Wilcoxon's *T*_(10)_ = 0.77, *p* = n.s.). EPI and structural images were coregistered and normalized to the ICBM152 standard template in Montreal Neurological Institute (MNI) space (resolution 2 × 2 × 2 mm^3^) using linear and nonlinear transformations and smoothed spatially with a Gaussian isotropic kernel of 6-mm full width half maximum. The functional data were filtered temporally using an autoregressive model (AR-1) and a high-pass filter with 171.5 s cut-off (corresponding to the duration of the longest game rounds).

#### Statistical analyses

We analysed our unconstrained video game playing data using event-related fMRI by focusing the analyses on win and loss events, whose timings were annotated automatically for every participant. Specifically, a random-effects model was implemented using a two-stage process. At the first level, each participant's hemodynamic responses to wins and losses during active and vicarious playing were modeled as delta (stick) functions, which were convolved with the hemodynamic response function (HRF). Joystick position and velocity time courses were included as nuisance regressors—head motion regressors were not included given that the motion adjustment procedure of ArtRepair toolbox (Mazaika et al., 2009) had already accounted for these. Individual contrast images for the conditions “winning while playing,” “winning while watching,” “losing while playing,” and “losing while watching” were then generated. At the second level, the first-level contrast images were subjected to a 2 (win vs. loss) ×2 (play vs. watch) factorial analysis, assuming dependency and unequal variances between the levels of both variables. With balanced designs at the first level (i.e., similar events for each subject, in similar numbers), this second-level analysis closely approximated a true mixed-effects design, with both within- and between-subject variance. At the second-level, we tested the main effects of contrasts “win > loss,” “loss > win,” “play > watch,” and “watch > play” with t-tests. To identify brain regions showing differential sensitivities to wins and losses during active and vicarious playing, we specified additional interaction contrasts “(play: win > loss) > (watch: win > loss)” and “(play: loss > win) > (watch: loss > win).” Statistical threshold in these analyses was set to family-wise error (FWE) corrected *P* < 0.05.

**Figure 2:**
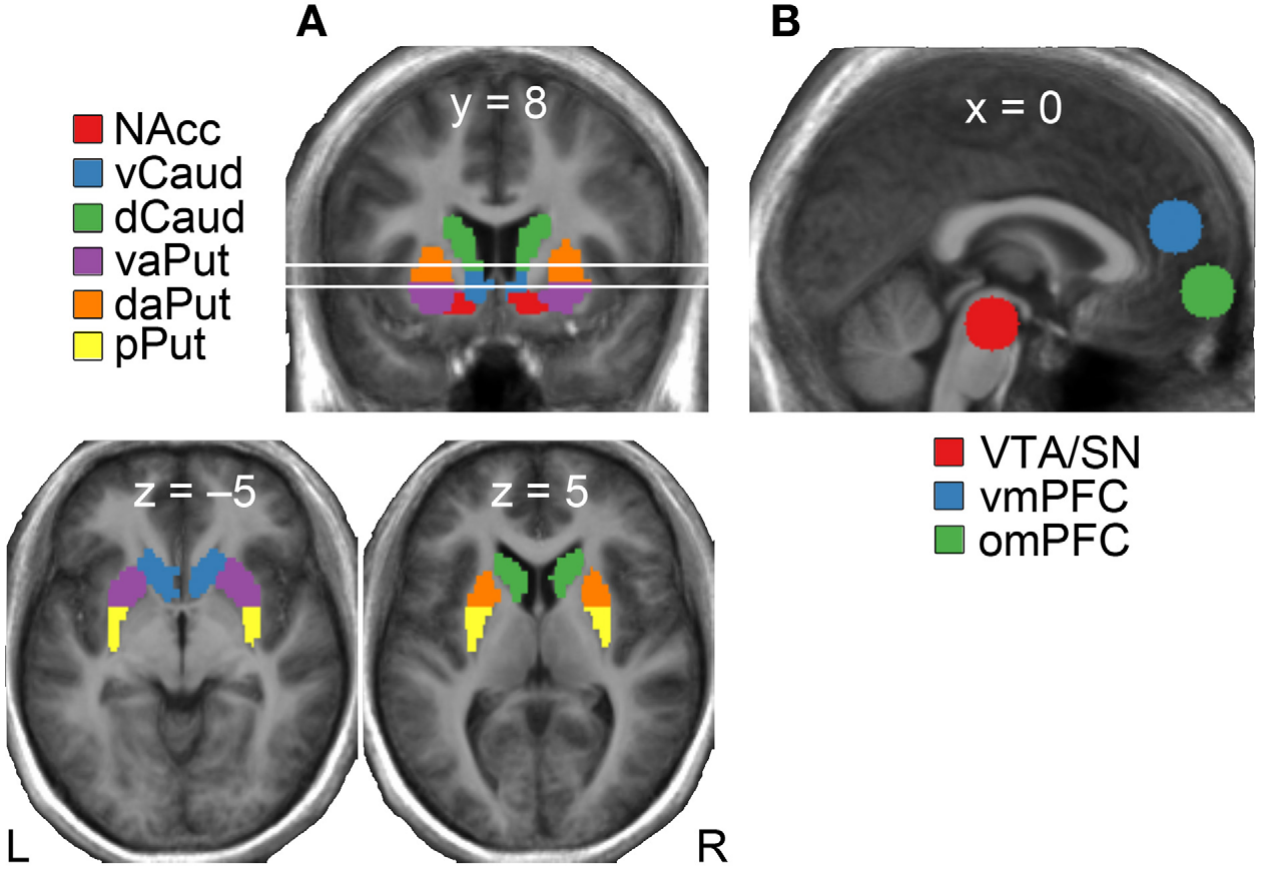
**Regions of interest (ROIs) in the (A) striatum, and (B) midbrain and frontal cortex**. NAcc, nucleus accumbens; vCaud, ventral caudate; dCaud, dorsal caudate; vaPut, ventral anterior putamen; daPut, dorsal anterior putamen; pPut, posterior putamen; VTA/SN, ventral tegmental area/substantia nigra; omPFC, orbitomedial prefrontal cortex; vmPFC, ventromedial prefrontal cortex.

We defined *a priori* regions of interest (ROI) for testing activations within mesial, striatal, and frontal parts of the reward circuit (Figure 2). Given that the striatum encompasses several anatomically and functionally segregated regions (cf. Haber and Knutson, 2010), we divided it into the following six subregions using the same classification as in our previous study (Kätsyri et al., 2012): nucleus accumbens (NAcc), ventral caudate (vCaud), dorsal caudate (dCaud), ventral anterior putamen (vaPut), dorsal anterior putamen (daPut) and posterior putamen (pPut). A spherical 10-mm ROI was defined for the VTA/SN (MNI coordinates 0, −22, −18) based on a previous study (O'Doherty et al., 2002). A spherical 10-mm ROI was derived for the vmPFC (MNI 0, 46, 18) based on a previous meta-analysis (Steele and Lawrie, 2004). Given that some fMRI studies on reward processing have reported more inferior reward-sensitive activation clusters, an additional 10-mm spherical ROI was extracted for the omPFC (MNI 0, 58, −6) from a previous study (Xue et al., 2009).

Correlations between self-ratings and mean beta responses in our predefined ROIs, during active versus vicarious playing, were tested with non-parametric Spearman's rank correlation tests. Correlations between pleasantness evaluations for specific game events and overall game experience evaluations were tested similarly. For these analyses, difference scores were first calculated between the playing and watching conditions for the pairs of variables in question, and correlations between them (*R*_Play-Watch_) were then tested. Given that such difference scores may produce spurious correlations (Cohen et al., 1983), we additionally calculated separate correlation coefficients for the variables constituting the difference scores (*R*_Play_ and *R*_Watch_) and set a criterion that their relative magnitudes should follow those of the difference scores (i.e., *R*_Play_ > *R*_Watch_ when *R*_Play-Watch_ > 0; and *R*_Play_ < *R*_Watch_ when *R*_Play-Watch_ < 0) for a difference score correlation to be considered as significant. Significance level thresholds for difference score correlations, when they were unplanned, were adjusted using false discovery rate (FDR) correction (Benjamini and Hochberg, 1995) at *P* < 0.05.

## Results

### Behavioral evaluations

Table 1 shows results from self-reports for active and vicarious playing conditions. Pleasantness ratings for wins and losses were significantly different from the scale's middle point (neutral emotional state) both during active (Wilcoxon signed rank tests: *Z* = 3.0 and −2.8, *P* = 0.003 and 0.004; effect sizes: *Pearson's R* = 0.64 and −0.61) and vicarious playing (*Z* = 2.9 and −2.9, *P* = 0.004 and 0.004, *R* = 0.62 and −0.61). Active versus vicarious playing did not differ with regard to number of wins (*R* = −0.31), number of losses (*R* = −0.35), or game end scores (number of wins minus losses; *R* = 0.01). These manipulation checks confirmed that players associated wins and losses with rewards and punishments, respectively, and that the numbers of wins and losses did not differ between active and vicarious playing conditions.

In contrast to the measures above, participants' experiences were clearly different during active and vicarious gameplay, with higher flow experience (*R* = 0.57), lower negative affect (*R* = −0.52), higher immersion (*R* = 0.49), and higher spatial presence (*R* = 0.57) during active playing. Similarly, players rated loss events as more unpleasant during active than vicarious playing (*R* = 0.57). Following the general guidelines of Cohen (1992), these results represent medium (*R* > 0.3) to large effect sizes (*R* > 0.5). Additionally, we observed borderline effects (*P* < 0.10) for higher challenge (*R* = 0.41) and higher positive affect (*R* = 0.38) during active playing with medium effect sizes. In contrast, players did not report significantly different social presence between active and vicarious playing (*R* = 0.08 for empathy, 0.32 for involvement, and 0.18 for negative feelings)—apparently, watching the game and playing it against an alleged computer-controlled opponent were associated with similar low levels of social presence.

We also tested whether participants' pleasantness evaluations for specific game events during active versus vicarious gameplay conditions were associated with their overall game experiences or BIS/BAS scores. The results showed that pleasantness difference scores for win events (active minus vicarious playing) were correlated positively with BAS fun seeking scores (*R*_Play-Watch_ = 0.79, *P* = 0.004, FDR-corrected *P*_thr_ = 0.010), and that the correlations for the constituent scores were meaningful (*R*_Play_ = 0.74 > *R*_Watch_ = −0.21). Significant difference score correlations were observed also between pleasantness evaluations for win events and competence, negative affect, and positive affect; however, these findings were rejected as spurious given that their constituent scores showed correlations whose relative magnitudes were opposite to expected.

### Full volume analysis of fMRI data

**Figure 3:**
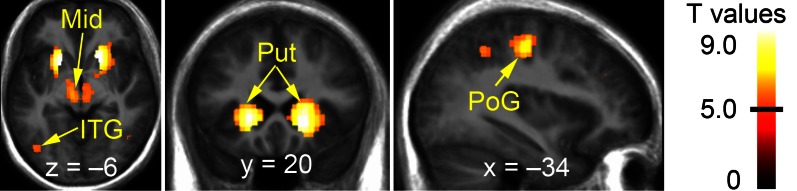
**Brain regions showing significantly stronger effects during vicarious than active playing (during win or loss gameplay events)**. The data have been thresholded at *P* < 0.05 (FWE-corrected; min. cluster size 50 voxels). Black horizontal line on the colorbar (on the right) illustrates the lowest significant *T*-value. Mid, midbrain; ITG, inferior temporal gyrus; Put, putamen; PoG, post-central gyrus.

Contrasting vicarious with active playing revealed activation clusters in the bilateral striatum, midbrain (including VTA/SN), sensorimotor cortices (pre- and postcentral gyri), and ventral visual stream (e.g., inferior temporal gyrus; Figure 3 and Table 2). To test whether these clusters reflected *activations during active playing* or *deactivations during vicarious playing* (or both), we defined contrasts for these effects (i.e., “watch+” and “play−”) and used them as implicit masks (*P* < 0.001) for the contrast between vicarious and active playing. All of the identified clusters in Table 2 survived implicit masking by deactivations during active playing (“play−”), whereas none of them survived implicit masking by activations during vicarious playing (“watch+”), confirming that the findings reflect systematic deactivations during active gameplay events.

**Table 2:** **Brain regions responding to vicarious versus active playing (pooled over both win and loss events)**.

**Region**	**Hemisphere**	**Voxels**	**Peak T**	**MNI coordinates**		
				***x***	***y***	***z***
Striatum (Put, Caud)	R	1553	10.99	18	20	−4
Striatum (Put, Caud)	L	1067	10.70	−18	14	−4
Pre- and postcentral gyri (BA3/4)	L	361	7.92	−34	−20	50
Midbrain (incl. VTA/SN)	L/R	560	7.10	2	−18	−10
Inferior parietal lobule (BA40)	R	93	6.96	54	−26	40
Inferior temporal gyrus (BA37)	L	125	6.95	−48	−70	0
Inferior parietal lobule (BA40)	L	54	6.32	−34	−48	46
Precuneus	R	66	6.03	28	−62	42

Activation clusters were thresholded at FWE-corrected P < 0.05 (minimum cluster size 50 contiguous voxels).

No significant activation or deactivation clusters were observed using the *a priori* significance threshold for the main effects of winning versus losing or vice versa, or for the interaction effects between wins versus losses and active versus vicarious playing or vice versa. However, using a small-volume correction for our *a priori* regions of interest (FWE-corrected threshold *P* < 0.05 at cluster-level) and a slightly more lenient threshold *P* < 0.001 (uncorrected) at voxel-level, we found stronger activations for wins versus losses in omPFC and bilateral ventral striatum. Furthermore, ventral striatal activations for wins versus losses were stronger during active than vicarious playing (Table 3). Using similar masking procedure as above, we found that even though wins evoked relatively stronger responses than losses during active gameplay, both events evoked BOLD signal decreases relative to the active gameplay baseline. Next we used detailed region-of interest analyses as described below to decompose these effects.

**Table 3:** **Brain regions showing statistically significant activation clusters after small-volume correction for all regions of interest**.

**Region**	**Hemisphere**	**Voxels**	**Peak T**	**MNI coordinates**		
				***x***	***y***	***z***
**WIN > LOSS**						
omPFC (medial frontal gyrus)	L/R	109	6.96	−2	56	−4
Striatum (Put, Caud)	R	362	5.28	24	10	−6
Striatum (Put, Caud)	L/R	246	5.08	−24	8	−4
**WIN > LOSS × PLAY > WATCH**						
Striatum (Put, Caud)	R	164	4.30	20	22	2
Striatum (Put, Caud)	L	72	3.81	−22	10	−4

Data were thresholded at FWE-corrected P < 0.05 (P < 0.001 at voxel-level).

### Region-of interest analysis in the reward circuit

We calculated mean beta values in our *a priori* ROIs and subjected them to analyses of variance (ANOVAs). First, we used omnibus analysis with 9 (Region: all striatal, frontal, and mesial ROIs) ×2 (Activity: playing, watching) ×2 (Event: win, loss) repeated-measures ANOVA to confirm that the following interactions with region were statistically significant: region × activity (*F*_(8, 80)_ = 12.08, *P* < 0.001, η^2^ = 0.07), region × event [*F*_(8, 80)_ = 8.89, *P* < 0.001, η^2^ = 0.06], and region × activity × event [*F*_(8, 80)_ = 3.45, *P* = 0.002, η^2^ = 0.01]. To break down these regional interactions, we conducted 2 (Activity) × 2 (Event) repeated-measures ANOVAs separately in all regions.

**Figure 4:**
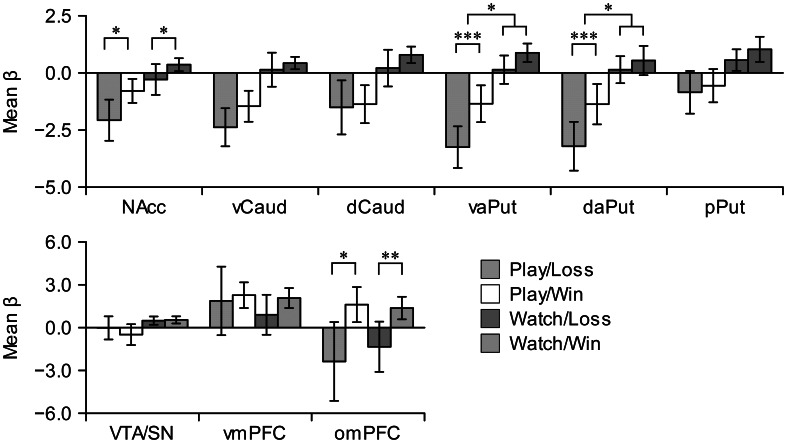
**Region of interest analyses in the striatal (upper row), and mesial and frontal nodes (lower row) of the reward circuit**. Error bars denote 95% confidence intervals. Asterisks denote significant simple effects (significant differences between wins vs. losses during either playing or watching) or significant interactions between game events and activities. ^*^P < 0.05. ^**^P < 0.001. ^***^P < 0.001. NAcc, nucleus accumbens; vCaud, ventral caudate; dCaud, dorsal caudate; vaPut, anterior ventral putamen; daPut, anterior dorsal putamen; pPut, posterior putamen; VTA/SN, ventral tegmental area and substantia nigra; vmPFC, ventromedial prefrontal cortex; omPFC, orbitomedial prefrontal cortex.

Figure 4 shows mean beta responses for win and loss events during active and vicarious playing conditions in all ROIs. Individual bar plots illustrate the activation directions (i.e., activations or deactivations) during win and loss events, and asterisks highlight significant differences between wins and losses. Wins versus losses evoked significantly greater effects, regardless of activity, in NAcc [playing: *F*_(1, 10)_ = 6.01, *P* = 0.03, η^2^ = 0.34; watching: *F*_(1, 10)_ = 6.14, *P* = 0.03, η^2^ = 0.31] and omPFC [playing: *F*_(1, 10)_ = 8.85, *P* = 0.014, η^2^ = 0.69; watching: *F*_(1, 10)_ = 24.77, *P* = 0.001, η^2^ = 0.71]. In contrast, wins versus losses evoked significantly greater effects only during active playing in vaPut [*F*_(1, 10)_ = 44.22, *P* < 0.001, η^2^ = 0.77] and daPut [*F*_(1, 10)_ = 70.08, *P* < 0.001, η^2^ = 0.81]. Interaction between action and event reached statistical significance in vaPut [*F*_(1, 10)_ = 8.09, *P* = 0.02, η^2^ = 0.03] and daPut [*F*_(1, 10)_ = 13.35, *P* = 0.004, η^2^ = 0.04]. The main effect of activity was significant in all striatal regions (*F*s > 11.45, *P*s < 0.007, η^2^ > 0.38) and, as can be seen in Figure 4, this clearly resulted from deactivations during active playing. Similar trend was evident also in VTA/SN [*F*_(1, 10)_ = 5.08, *P* = 0.048, η^2^ = 0.23]. Taken together, these results demonstrate that although both win and loss events elicited deactivations in the striatum during active playing, activations in NAcc and aPut (both vaPut and daPut) returned closer to baseline levels during win events; furthermore, wins versus losses evoked greater activation changes in aPut during active than vicarious playing. Inspection of individual mean beta responses demonstrated that the latter result was robust; that is, mean beta responses to wins versus losses in aPut were greater during active than vicarious playing with nine out of eleven participants. In contrast to the aPut region, omPFC showed greater activations during win events regardless of active and vicarious playing.

### Correlations between behavioral and fMRI responses

We predicted that the players' self-evaluations for pleasantness of wins versus losses during active versus vicarious playing would be associated with the corresponding BOLD signal changes in the striatum. To test this hypothesis, we calculated differences between wins and losses during active versus vicarious playing [i.e., contrast “(play: win > loss) > (watch: win > loss)”] both for pleasantness ratings and mean beta values. Contrary to our predictions, no statistically significant correlations between these variables were found in any striatal region (*R*s < 0.51; *P*s > 0.11). Similarly, we failed to find statistically significant correlations between pleasantness ratings and mean beta values for wins versus losses, pooled over active and vicarious playing, in either of the frontal ROI (*R*s < 0.18, *P*s > 0.59).

**Figure 5:**
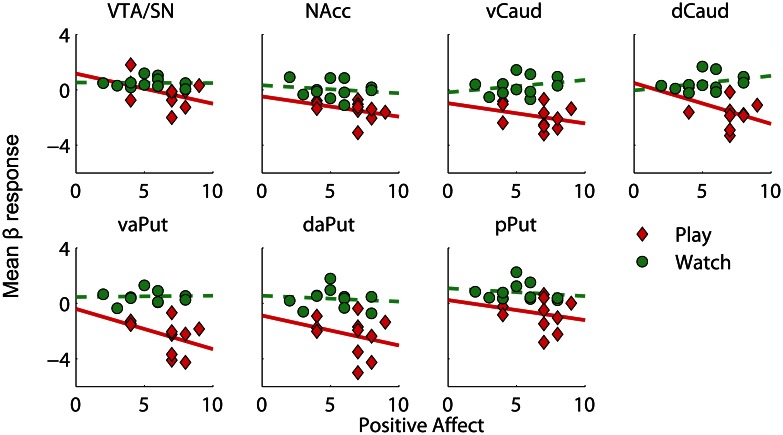
**Bivariate scatter plots for Positive Affect evaluations versus mean beta responses in regions of interest, for active and vicarious playing conditions**. The solid and dashed lines depict best linear fits to the data. VTA/SN, ventral tegmental area and substantia nigra; NAcc, nucleus accumbens; vCaud, ventral caudate; dCaud, dorsal caudate; vaPut, anterior ventral putamen; daPut, anterior dorsal putamen; pPut, posterior putamen.

One possibility for explaining the systematic deactivations in midbrain and striatum during active versus vicarious playing (cf. Figure 4) is that their activations remained elevated throughout the active gameplay due to anticipatory or hedonic reward processing but returned closer to baseline levels during both win and loss events. To test this, we calculated difference scores between active and vicarious playing for positive and negative affect measures, and compared them against mean beta values for active versus vicarious playing (pooled over wins and losses) in our predefined ROIs. Consistently, positive affect difference scores showed a significant correlation with deactivation strengths in VTA/SN, dCaud, and vaPut, and a marginally significant correlation with deactivations in vCaud (Table 4); all of these effects were large (*R* > 0.50). When calculated separately, correlation coefficients in these ROIs were more negative during active than vicarious playing (i.e., *R*_Play_< *R*_Watch_). Bivariate scatter plots for these correlations are shown in Figure 5. In other words, the greater deactivations these regions exhibited during the win and loss gameplay events, the higher positive affects the players reported after active than vicarious playing.

**Table 4:** **Correlations between difference scores (active minus vicarious playing) for positive and negative affect measures, and mean beta values (active versus vicarious playing) in mesial and striatal regions (*R*_**Play–Watch**_)**.

**Region**	**NA**				**PA**			
	***R***_**Play–Watch**_	***R***_**Play**_	***R***_**Watch**_	***P***_**Play–Watch**_	***R***_**Play–Watch**_	***R***_**Play**_	***R***_**Watch**_	***P***_**Play–Watch**_
NAcc	−0.06	0.05	−0.27	0.872	−0.60	−0.58	−0.09	0.054
vCaud	−0.04	−0.08	−0.26	0.914	−0.63	−0.27	0.18	0.037^†^
dCaud	0.12	0.21	−0.17	0.726	−0.81	−0.35	0.40	0.002^*^
vaPut	0.12	−0.19	0.27	0.715	−0.74	−0.50	−0.04	0.010^*^
daPut	0.18	−0.31	0.52	0.607	−0.45	−0.21	−0.18	0.163
pPut	0.15	−0.35	0.48	0.665	−0.48	−0.22	−0.34	0.132
VTA/SN	0.53	0.17	0.78	0.094	−0.70	−0.15	0.01	0.016^*^

Separate correlation coefficients for active and vicarious conditions (*R*_Play_ and *R*_Watch_) are also displayed to facilitate the interpretation of difference score correlations. Significance values are from Spearman's rank correlation tests.

† P < 0.05 (uncorrected).

* P < 0.05 (FDR-corrected).

## Discussion

In the present investigation we studied fMRI responses to win and loss gameplay events (relative to activation levels during generic video game playing) during active and vicarious gameplay. Our results revealed two main effects in the striatum. First, replicating similar previous findings (Mathiak et al., 2011), both win and loss events evoked *deactivations* with respect to generic gameplay levels during active but not during vicarious playing. Second, in addition to this main effect of gameplay activity, win events evoked higher activation levels (i.e., weaker deactivations during active playing and stronger activations during vicarious playing) than loss events. Furthermore, our results showed an interaction between these two effects; that is, activation changes due to wins versus losses in the striatum, particularly in the anterior putamen, were larger during active than vicarious playing. This interaction effect demonstrates for the first time that winning versus losing in a complex video game evokes stronger effects in the striatum during active than vicarious gameplay. This finding is consistent with both animal electrophysiology (Kawagoe et al., 1998; Schultz et al., 2000) and human neuroimaging (Elliott et al., 2004; O'Doherty et al., 2004; Tricomi et al., 2004; Zink et al., 2004; Guitart-Masip et al., 2011), showing that striatal reward responses depend critically on the recipients' own actions. These previous studies have employed simple tasks where rewards were associated with specific motor actions (e.g., pressing one of two buttons), whereas the present study extends these findings by demonstrating action-reward contingency in the striatum during a complex, ecologically valid task (video game playing) that simulates free-ranging human motivated behavior.

We were also able to dissociate the coding of actively versus passively obtained rewards in the striatum and frontal cortex: whereas the anterior putamen was more sensitive to wins than losses only during active gameplay, the omPFC showed stronger activation to winning than losing during both active and vicarious playing. Action-independent reward activations in the omPFC have been observed previously in both animal (Schultz et al., 2000) and human neuroimaging studies (Elliott et al., 2004). Given that the win and loss events were associated with external monetary rewards and punishments, the omPFC activations are also consistent with the known role of omPFC in processing monetary gains and other secondary rewards (Xue et al., 2009). However, nucleus accumbens in the striatum also showed greater activations to wins than losses during both active and vicarious gameplay. It is possible that, unlike the anterior putamen, nucleus accumbens was generally sensitive to receiving rewards similarly as omPFC. The dissociable response patterns of anterior putamen and nucleus accumbens results could stem from the different connectivity patterns of ventromedial striatum (including nucleus accumbens) and dorsolateral striatum (including putamen): whereas the ventromedial striatum receives visceral afferents, the more dorsolateral regions are connected predominantly with higher-order associative and sensorimotor regions (Voorn et al., 2004).

The observed striatal deactivations during both wins and losses during active playing significantly extended our previous findings (Kätsyri et al., 2012). Although similar striatal deactivations have been observed previously (Mathiak et al., 2011), reward circuit deactivations associated with rewarding gameplay events nevertheless warrant consideration. One possible explanation is that the striatum showed tonic activations when the player was actively competing against his opponent, and that these activations returned closer to baseline levels whenever a break in the game restrained him from pursuing this goal; that is, both after he became incapacitated (loss events) and after he managed to eliminate his opponent (win events). Unfortunately, we were not able to test this hypothesis directly: as the strength of a raw BOLD signal is arbitrary, comparing the intercepts of independently scanned active and vicarious playing sessions would have been nonsensical. However, previous fMRI and PET studies have already demonstrated that active gameplay evokes tonic increases in striatal activations (Koepp et al., 1998; Hoeft et al., 2008), and one previous study has shown that active gameplay onsets and offsets evoke striatal fMRI activations and deactivations, respectively (Cole et al., 2012).

Above we suggested that striatal deactivations taking place at the times of wins and losses could be caused by tonic activation levels during generic gameplay, which returned closer to baseline levels when the gameplay activity was interrupted. Although this suggestion is speculative, there are at least two potential explanations for why video game playing would evoke tonic activations in the striatum. First, such activations, particularly during active playing, could reflect the inherently rewarding nature of playing *per se* (cf. Przybylski et al., 2010; see also Koepp et al., 1998). Our results tentatively support this view, given that the striatal and mesial deactivations caused by gameplay events (wins and losses) during active versus vicarious gameplay were correlated with the players' positive affect self-ratings for the corresponding whole sessions. Second, it is possible that the tonic striatal activations would reflect sustained anticipatory rather than hedonic reward processes—that is, ‘wanting’ rather than ‘liking’ components of reward (see Berridge, 2007; Diekhof et al., 2012). This is a plausible explanation, given that in our fast-paced video game (with 20–30 s. mean round durations; see Table 1), all activities following the onset of a new game round (i.e., finding and engaging the opponent) were ultimately associated with reward seeking. It is, however, uncertain why such anticipatory responses should be greater during active than vicarious playing. Furthermore, anterior putamen (during active playing) and nucleus accumbens (during both active and vicarious playing) were sensitive also to reward outcomes, as their responses were greater for wins than losses. The suggestions on striatal responses to anticipated and obtained rewards are not mutually exclusive. In fact, a recent meta-analysis of brain imaging studies demonstrated that the ventral striatum, unlike mPFC, is sensitive to both anticipated and received rewards (Diekhof et al., 2012).

In addition to affective evaluations, active and vicarious playing conditions evoked differential spatial presence and flow experiences. Spatial presence has been associated with activations in a wide network including the ventral visual stream, the parietal cortex, the premotor cortex, and the brainstem (Jäncke et al., 2009). Interestingly, our results demonstrated that in addition to the striatum, also these regions showed strong deactivations during win and loss events during active playing (cf. Table 2). Hence, it is possible that also the network contributing to the experience of spatial presence showed tonic activations during active playing, which returned to baseline levels after win and loss events. Nevertheless, it is clear that future studies are needed for disseminating the tonic and phasic fMRI activations and their behavioral correlates (e.g., spatial presence) during video game playing.

In line with the attribution theory (Weiner, 1985), players' self-ratings confirmed that losses were experienced as more unpleasant during active than vicarious playing, even though the external monetary rewards and punishments for wins and losses were identical during active and vicarious playing conditions. The perceived pleasantness of win events during active playing was also linked to individual differences in appetitive motivation (i.e., tendency for fun seeking). Our results nevertheless did not provide evidence for associations between players' pleasantness self-ratings and their fMRI responses to win and loss events in general, or between the active and vicarious playing activities. However, it should be noted that players made only two evaluations with respect to all the win and loss events of a game, respectively, and it is possible that such overall evaluations may not have been as accurate as *post-hoc* evaluations for all game events would have been. In future, this problem could be solved by showing participants video recording of their gameplay sessions, and asking them to continuously rate their emotional feelings during the gameplay; this technique has been proven successful for example when studying the brain basis of emotions elicited by movies (Nummenmaa et al., 2012) and already utilized in previous fMRI game studies (Klasen et al., 2008).

Our subjects used precision hand actions to manipulate the joystick, and thus it is critical to control for sensorimotor processes related to the acquisition of rewards, especially because the striatum is also involved in sensorimotor control over corrective hand movements (Siebner et al., 2001; Turner et al., 2003). This issue is particularly important for win and loss events, given that these events are typically followed by different changes in movement demands (e.g., continuation of gameplay vs. total immobility). To our knowledge, however, previous brain imaging studies have not explicitly tried to control for joystick movement confounds. Even after we included continuous confound regressors both for overall movements and for movement direction changes, our results clearly demonstrated similar striatal effects for video game playing events as previously reported (Klasen et al., 2012), implying that such results cannot be accounted for by sensorimotor effects. Nevertheless, the effects of varying movement demands following win and loss events could be studied more explicitly in the future; for example, by manipulating whether the player is able to move after specific game events or not. Future studies with explicit focus on testing the role of reward anticipation versus reward reception in striatal responses should also be conducted. Such studies should utilize slower-paced video games with sufficiently long durations between critical actions (such as shooting) and their outcomes.

Although the present sample size was comparable to those of several recent fMRI studies utilizing video game stimuli (Mathiak and Weber, 2006; Weber et al., 2006; Mobbs et al., 2007; Ko et al., 2009; Mathiak et al., 2011; Klasen et al., 2012), future studies should consider using larger sample sizes to detect potentially more fine-grained differences between active and vicarious gameplay. We conducted retrospective power analysis for our data using G^*^Power software (Faul et al., 2007) to estimate the minimum sample sizes that should be used in future within-subjects studies to detect similar effects with 80% statistical power (at 5% significance level). These calculations showed that five participants would be sufficient for detecting similar win versus loss responses in the ventral anterior putamen (*M* = 1.90, *SD* = 0.95, and γ = 2.0). However, to replicate the differential win versus loss responses during active versus vicarious playing in the same region, a larger sample of at least thirteen participants should be used (*M* = 1.16, *SD* = 1.35, γ = 0.86). As the present study has demonstrated, automatic annotation of gameplay events allows easy acquisition of large datasets from naturalistic video game playing tasks.

In conclusion, we have shown, utilizing novel video-game-playing tasks, that striatal and frontal dopaminergic reward circuit nodes respond to wins and losses differentially during active and vicarious gameplay. Specifically, the striatal node (in particular, anterior putamen) was more sensitive to wins than losses only during active game playing, whereas the frontal node (omPFC) showed stronger responses to wins than losses regardless of activity. These results highlight the role of the striatum in encoding self-acquired versus passively obtained rewards during free-ranging motivated behavior. Although the audiovisual stimulation provided by modern video games may be rewarding by itself, the neural underpinnings of hedonic and aversive experiences during video game playing clearly depend also on the players' active engagement in the game. The striatal reward processing circuitry explored in the current study likely contributes to the motivational pull of video-game playing.

### Conflict of interest statement

The authors declare that the research was conducted in the absence of any commercial or financial relationships that could be construed as a potential conflict of interest.
